# On the switching dynamics of epitaxial ferroelectric CeO_2_–HfO_2_ thin film capacitors

**DOI:** 10.1186/s40580-022-00344-4

**Published:** 2022-12-14

**Authors:** Felix Cüppers, Koji Hirai, Hiroshi Funakubo

**Affiliations:** 1 Department of Materials Science and Engineering, Tokyo Institute of Technology, 226-8502 Yokohama, Japan; 2 PGI-10, Forschungszentrum Jülich GmbH, Jülich, Germany

## Abstract

Epitaxial layers of ferroelectric orthorhombic HfO_2_ are frequently investigated as model systems for industrially more relevant polycrystalline films. The recent success in stabilizing the orthorhombic phase in the solid-solution cerium oxide – hafnium oxide system allows detailed investigations of external influences during fabrication. This report analyzes the ferroelectric properties of two thin film capacitors, which were post-deposition annealed in N_2_ and O_2_ atmospheres to achieve the orthorhombic phase after room temperature deposition. The samples, which exhibit very similar constituent phase, appear identical in conventional polarization-field hysteresis measurements. However, a significant switching speed difference is observed in pristine devices. Continued field cycling reduces the difference. Deeper analysis of switching transients based on the Nucleation Limited Switching model suggests that the O_2_ heat treatment atmosphere results in an altered oxygen vacancy profile, which is reverted during ferroelectric cycling.

**Supplementary Information:**

The online version contains supplementary material available at 10.1186/s40580-022-00344-4.

## Introduction

Thin films of HfO_2_ have received widespread attention in research and industry since their adoption as high-k gate dielectric [1]. Various properties of the material have been discovered which have opened new application options, such as flash memory [2], dynamic random access memories, DRAM [3] and memristive functions [4, 5].

In 2011, ferroelectricity in Si-doped HfO_2_ thin films was reported [6] and in 2015 the first successful epitaxial deposition of Y-doped HfO_2_ was published [7]. Since then, significant research efforts have been ongoing, both on the technological side as well as the fundamental understanding. HfO_2_ thin films show pronounced polymorphism between the monoclinic (m-), tetragonal (t-), cubic (c-), rhombohedral (r-) and orthorhombic (o-) phases [8–14]. A major concern in ferroelectric research is therefore the stabilization of the appropriate phase, which is typically the noncentro-symmetric o-phase *Pca*2_1_, although recent reports show ferroelectric properties in r-phase as well [13, 15]. Various approaches have been studied such as specific deposition control [16, 17] targeted heat treatment [18–20] and doping with elements [21–23], among others. A typical approach to study the fundamental properties of ferroelectricity in HfO_2_ is to eliminate the influence of microstructure as much as possible, i.e. epitaxial growth. Substrate selection and lattice matched bottom electrodes are essential in this approach. Combined with careful dopant control and targeted heat treatment, epitaxial thin films with high desired phase contents are obtained. Such samples are ideal candidates to study effects which are otherwise inaccessible because of possible phase transformations during cycling or multi-grain influences. An open question for HfO_2_ ferroelectrics is the effect of oxygen vacancy concentration on the switching dynamics. The approach of this study is to isolate the effect of oxygen vacancy concentration from the phase purity and orientation purity by utilizing epitaxially grown films on lattice matched bottom electrodes.

Therefore, in the present study, the influence of gas atmosphere during the commonly performed heat treatment step with continued field cycling on the ferroelectric switching speed is investigated. For this purpose, the recently reported solid-solution epitaxial system of (111)-oriented 17% CeO_2_–83% HfO_2_, which shows the highest fraction of o-phase in the composition range, is chosen as model system [24]. Because of the (111) In_2_O_3_-SnO_2_ (ITO) bottom electrode and the precise doping, the o-phase is predominant irrespective of the employed gas atmosphere during the heat treatment. The results of this letter reveal that the heat treatment gas atmosphere has a significant effect on the switching speed in pristine samples in this novel material system. The influence diminishes during cycling of the capacitors. Because of the epitaxial nature of the films, we propose that generated and redistributed defects are the leading cause for the switching speed change. It is found that the rate limiting step transitions from mainly domain wall motion limited to nucleation limited.

## Experimental methods

The sample fabrication is described in detail in the recent report on the composition dependence of the (Hf_1 − *x*_Ce_*x*_)O_2_ system [24]. In the scope of this study, only the *x* = 0.17 samples are considered since they provided the highest o-phase fraction after deposition. Importantly, the post-deposition annealing conditions were identical to [24], i.e. rapid thermal annealing before top electrode fabrication at 1000 °C for 10 min with heating and cooling rate of 25 °C/s and 8 °C/s, respectively. Atmospheric oxygen and nitrogen were employed through constant gas flows of 100 cm^3^/min and 100 cm^3^/min, respectively. The final stack of the samples is (111)YSZ// 50 nm-(111)ITO// 20 nm-Hf_0.83_Ce_0.17_O_2_ // 100 nm-Pt. The ferroelectric film thickness of 20 nm is required for high signal-to-noise ratio during phase identification using Reciprocal Space Mapping as described in our previous report. The properties of thinner layers are currently under investigation. The measured capacitors in this study were all 50 μm in diameter.

For electrical testing, a ferroelectric test setup (Toyo FCE-1) with a Toyo Corporation HVA-300 module was employed. This setup allows to measure current transients with a sampling rate of 250 million data points per second.

## Results and discussion

### Results

**Fig. 1:**
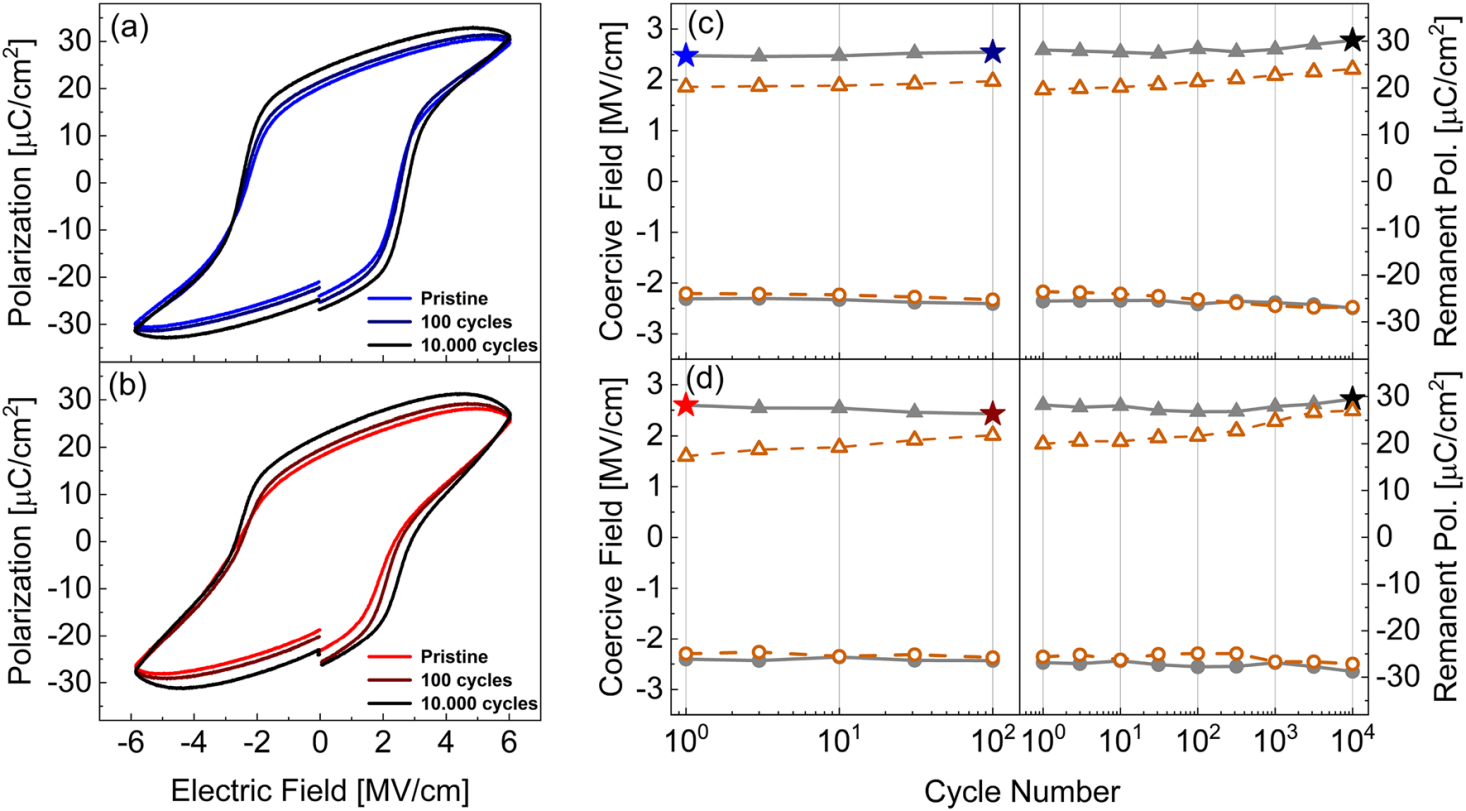
**a**, **b** Polarization-field hysteresis loops and **c**, **d** cycling experiment for the sample annealed in N_2_ atmosphere and sample annealed in O_2_ atmosphere, respectively. Coercive fields are depicted as grey closed symbols, remanent polarizations as orange open symbols. Coercive fields at measured cycling stages are indicated as star symbols

Figure 1 shows the polarization (*P)*–electric field (*E*) loops measured using bipolar triangular pulses of both the N_2_ annealed (a) and the O_2_ annealed (b) samples. *P*–*E* hysteresis were recorded at 10 kHz frequency and are average of 3 cycles each. Both samples show clear ferroelectric property in the pristine state, without wake-up process. This feature is different from reported polycrystalline samples [25–27], but is frequently observed in epitaxial films [13, 28–30] and may be due to the high phase and orientation purity of lattice-matched epitaxial films. The coercive field, *E*_c_, is nearly symmetrical for up and down polarization switching at approximately |2.5 MV/cm|. While polycrystalline samples typically show lower *E*_c_, it is a common value for epitaxial films in this thickness range [9]. The investigation on the effect of thickness scaling in this dopant system is ongoing. Figure 1c and d shows the two-staged cycling experiment of each sample. Polarization cycles were realized as 100 kHz rectangular signals of ± 6 MV/cm. Coercive fields of the *P*-*E* measurements are plotted as grey closed symbols, while the values of remanent polarization *P*_r_ are depicted as open orange symbols. Selected values of the coercive field for positive polarization reversal are highlighted as star symbols. The according *P*-*E* loops are plotted in Fig. 1a and b. The lack of wake-up in these samples is visible, as *P*_r_ remains high throughout the measurement. A slight asymmetry in remanent polarization is observed, which may be related to the asymmetric electrodes of the stack.

**Fig. 2:**
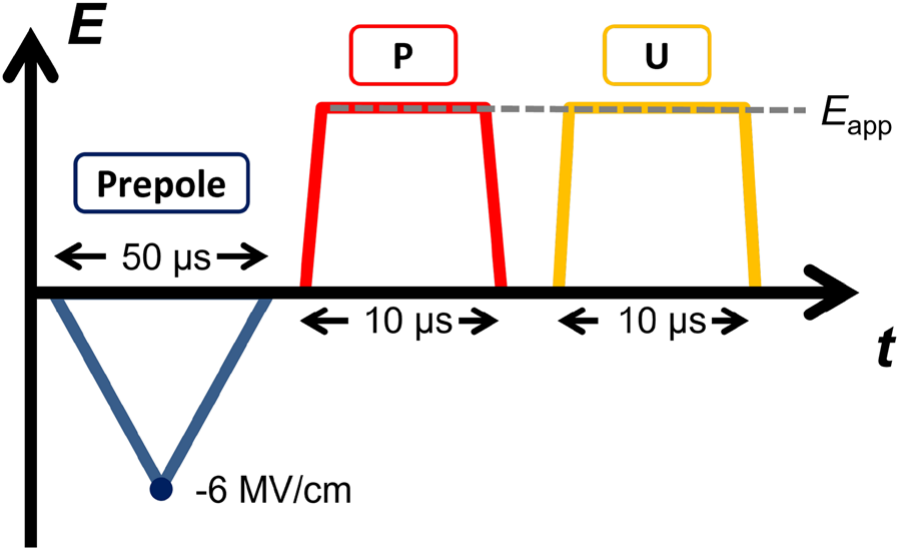
Prepole-Positive-Up measurement scheme

Both samples showed similar endurance to around 10^6^ cycles at 100 kHz frequency. The subsequent failure was not preceded by fatigue symptoms such as diminishing *P*_r_, but appeared spontaneously. In contrast, *P*_r_ seems to increase during the measurement. However, the negative slope of *P* as the field approaches the maximum/minimum indicates an increase in leakage current, which artificially increases the value of *P*_r_. The following prepole-P-U analysis (See Fig. 2) revealed that the value of *P*_r_ actually remains nearly constant between the pristine sample and after 10^4^ cycles.

**Fig. 3:**
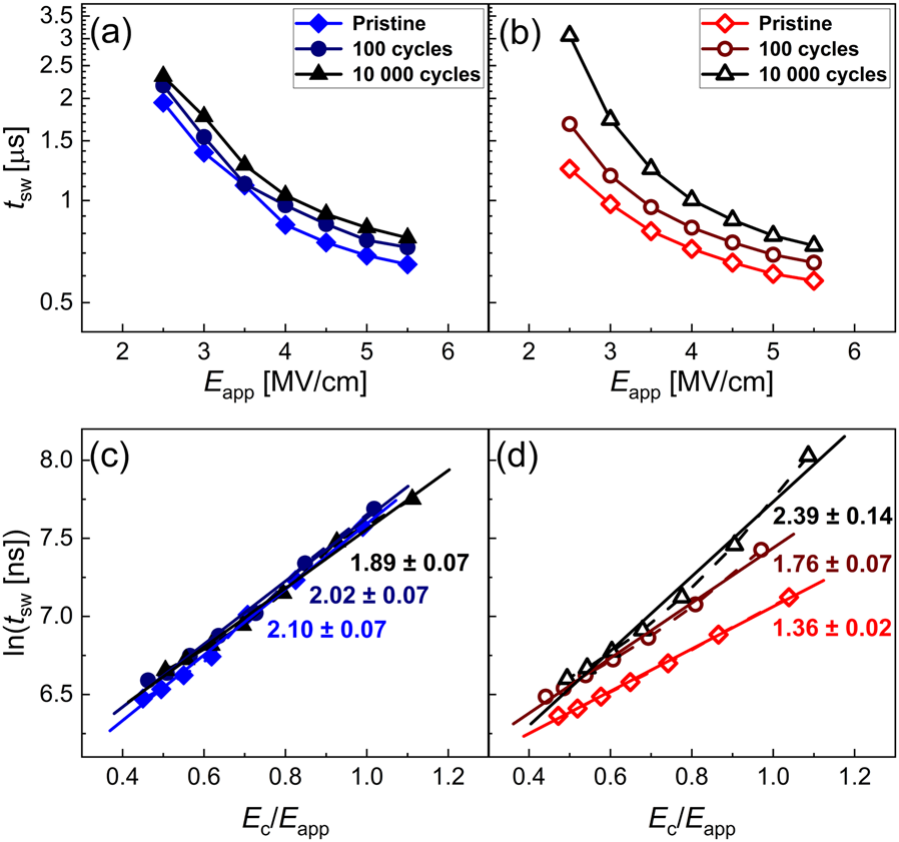
**a** and **b**: Calculated switching times from the measured polarization transient curves for different cycling stages of the sample treated in N_2_ and O_2_, respectively. **c** and **d**: Extraction of activation field versus coercive field ratio from the modified Merz’ law of Eq. (3) of the sample treated in N_2_ and O_2_, respectively

To study the switching kinetics of the two samples annealed under N_2_ and O_2_ atmosphere, the measurement procedure shown in Fig. 2 is employed. It consists of three distinct parts. During the prepole signal, the device is subjected to a 50 µs long triangular voltage signal to -6 MV/cm. The following positive (P) signal is of 10 µs duration and variable field. After a 5 µs delay, the same signal shape is applied once more as the up (U) signal. Similar to the PUND sequence [31], this measurement technique allows subtracting the leakage current and the dielectric charging component, recorded during the U pulse, from the P pulse, which also contains the possible polarization reversal current. Hence, by integration of the subtracted current signal the polarization charge density

1 $${\varDelta P}_{sw} (t)=\int_{0}^{10\mu s} (I_P-I_U) dt$$

can be calculated. To exclude the influence of the rising voltage, only currents after 90% of applied electric field (*E*_app_) is reached are considered. The switching time *t*_sw_ is defined as the time for reaching 50% of the achievable polarization for the given field. Figure 3a and b show *t*_sw_ versus *E*_app_ at different cycling stages for the samples annealed under N_2_ and O_2_ atmosphere, respectively. Both samples show a clear dependence of *t*_sw_ on *E*_app_ as expected. Furthermore, the switching speed appears to be dependent on the cycling stage for both samples. The increase in *t*_sw_ is more significant for the sample annealed in O_2_, which shows faster switching than the N_2_ annealed sample in the pristine state.

As seen in Fig. 1, *E*_c_ changes during the cycling. The comparison of switching transients should therefore be considered with respect to *E*_app_ normalized by *E*_c_, i.e. the fraction (*E*_app_ / *E*_c_) at the different cycling stages. Merz’ law [32] is a good descriptive model for the *t*_sw_ versus *E*_app_ relation. It can be modified to incorporate the consideration of non-constant *E*_c_:

2 $$t_{{sw}} = \tau _{0} {\text{ exp}}\left( {\frac{{E_{A} }}{{E_{{app}} }}} \right) = \tau _{0} {\text{ exp}}\left( {\frac{{E_{A} }}{{E_{c} }} \cdot \frac{{E_{c} }}{{E_{{app}} }}} \right)$$

and rewritten into the linear form

3 $$\text{ln}\left({t}_{sw}\right)=\text{ln}\left({\tau }_{0}\right)+\frac{{E}_{A}}{{E}_{c}}\cdot \frac{{E}_{c}}{{E}_{app}}$$

Here, *E*_A_ is the activation energy for polarization reversal and τ_0_ the theoretical switching time for infinitely strong fields.

Figure 3c and d show the Merz’ law fits for the sample annealed under N_2_ and O_2_ atmosphere, respectively. The solid lines show linear least-squares fits to the data points. Inset values are the fit slopes, (*E*_A_/*E*_c_). The slope and position of the N_2_ annealed sample remains constant for the cycling stages. The slope of the O_2_ annealed sample shows significant shift towards higher values of (*E*_A_/*E*_c_) for the cycling stages, indicating internal changes in the film.

The differences in switching kinetics are further examined by comparing the recorded polarization transients to common switching models. This approach has been employed before for HfO_2_ ferroelectric systems [33], although in most publications discrete probing pulses are used [34–36]. Regarding the appropriate model, a wide variety has been proposed in literature, including the Kolgomorov-Avrami-Ishibashi (KAI) model [37–39], Inhomogeneous-Field-Mechanism (IFM) model [40, 41], multi-grain Landau-Khalatnikov (LK) model [42] and the Nucleation-Limited-Switching (NLS) model [43–47]. In the scope of this study, we rule out both the IFM and the multi-grain LK model due to their nature of assuming a polycrystalline film with wide variety of polarization orientations, which is the opposite to our highly oriented epitaxial films [24]. The KAI model is considered appropriate for epitaxial films in classical ferroelectric materials since it describes homogeneous domain nucleus formation and unhindered domain wall motion as the limiting step similar to high-purity bulk materials [43, 48, 49]. However, multiple recent studies have demonstrated that the NLS model is a better fit for HfO_2_ thin films [34, 35, 50, 51]. This model assumes inhomogeneous formation of nuclei in different regions of the film with a characteristic distribution. Mathematically, the ferroelectric response of the NLS model is defined as

4 $${\varDelta P}_{sw}\left(t\right)=2{P}_{s}\cdot {\int }_{\!\!\!\!-\infty }^{\infty }\left[1-\text{e}\text{x}\text{p}\left\{{-\left(\frac{t}{{t}_{0}}\right)}^{n}\right\}\right]\cdot F\left(\text{log}\left({t}_{0}\right)\right) d\left(\text{log}\left({t}_{0}\right)\right)$$

Here, *P*_s_ is the switchable polarization, *t*_0_ is a characteristic switching time and *n* is the domain expansion coefficient. *F*(log(*t*_0_)) denotes a Lorentzian distribution function, which describes the assumed spread of characteristic switching times of individual domains:

5 $$F\left(\text{log}\left({t}_{0}\right)\right)=\frac{A}{\pi }\left[\frac{w}{{\left(\text{log}\left({t}_{0}\right)-\text{log}\left({t}_{1}\right)\right)}^{2}+{w}^{2}}\right]$$

Here, *A* is a normalization constant, *w* is the half width at half maximum of the distribution, and *t*_1_ is the distribution center. The transient polarization curves were fitted using an interior-point algorithm that was allowed to vary the parameters [*P*_s_, *n*, *t*_1_, *w*] to minimize the total squared error between experimental data and fit. A rough parameter guess was supplied to the algorithm to ensure quick convergence and to reduce the risk of convergence to sub-optimal local function minima. The minimum *R*^2^ value of the fit to the experiment is 0.933 and the mean *R*^2^ value of all fitted curves is 0.992, indicating very accurate matching of the above NLS equations to the experimental data. The value of *P*_s_ converged very closely to the polarization at *t* = 10 µs, as expected. The value of *n* is between 2 and 3, which indicates 2- to 3- dimensional domain growth.

**Fig. 4:**
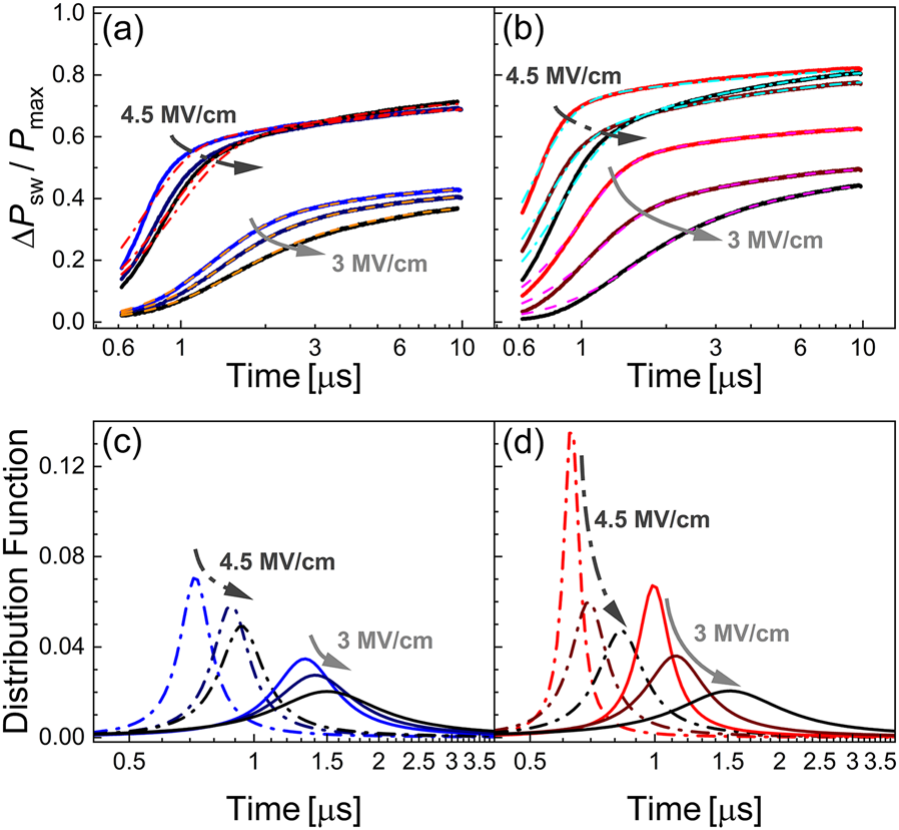
**a** and **b**: Measured and NLS fit polarization transient curves for different cycling stages of the sample treated in N_2_ and O_2_, respectively. **c** and **d**: Corresponding distribution functions of the characteristic switching times

The solid lines in Fig. 4a and b show the transient polarization curves as calculated by Eq. (1) for the N_2_- and the O_2_-treated samples, respectively. For readability, three cycling stages of two exemplary fields, 3 MV/cm and 4.5 MV/cm, are shown. The curves are normalized to the maximum reached polarization at the given cycling stage. The corresponding NLS model fits are shown as dashed lines. The difference of the cycling stages, which are indicated by the direction of the arrows, is visible both in switching magnitude and speed from the transients. The O_2_ sample shows a more pronounced speed lowering.

Figure 4c and d show the Lorentzian distributions of Eq. (5) for the fits in (a) and (b), respectively. Arrows indicate the increase in cycling stage. Both samples exhibit a lower center position and wider distribution. The effect is much more pronounced for the O_2_-treated sample. The distributions after 10^4^ cycles look almost identical for both samples.

**Fig. 5:**
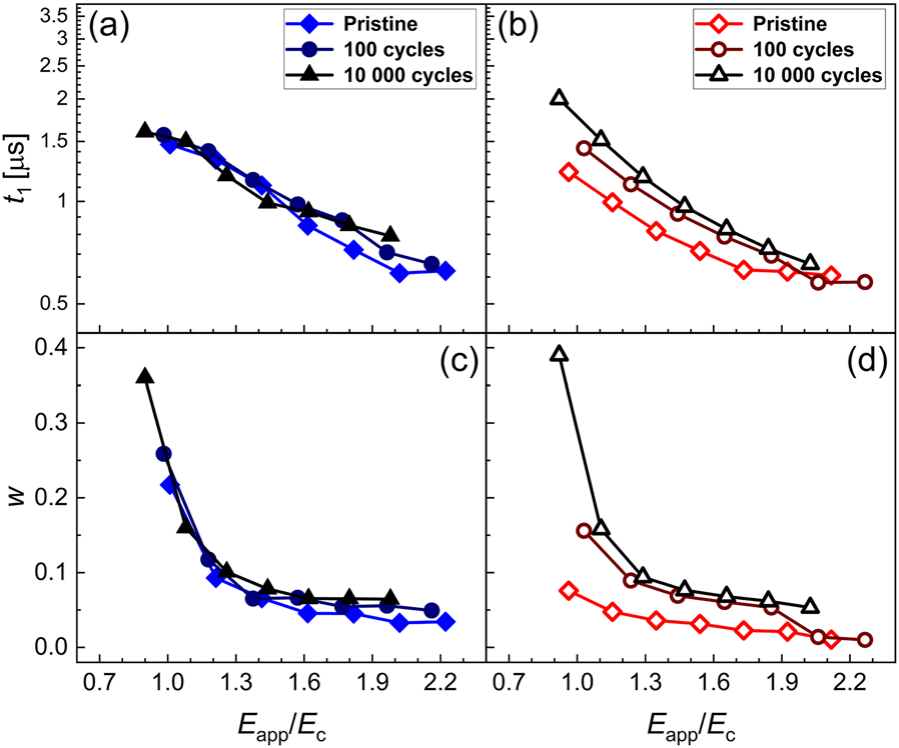
**a** and **b**: NLS fit parameter *t*_1_. **c** and **d**: NLS fit parameter *w* of the sample treated in N_2_ and O_2_, respectively

The parameters *t*_1_ and *w*, which determine the shape of the characteristic switching time distribution, are shown for both samples respectively in Fig. 5. Since the ratio of (*E*_app_/*E*_c_) reveals a difference in the internal behavior as described above, this approach is chosen here. While the N_2_ sample shows very minor dependence on the cycling stage, the O_2_-annealed sample changes significantly during cycling. Both *t*_1_ and *w* increase with increasing switching cycles. The change of *t*_1_ is very similar to the change in *t*_sw_ shown in Fig. 3b, which is due to the low values of *w*.

### Discussion

The slight change in switching speed of the N_2_-treated sample, see Fig. 3a can be explained mainly by considering the according change in the coercive field. There are multiple possible explanations for changes in *E*_c_. Apart from the frequently argued domain wall pinning mechanisms [52, 53], other effects such as local imprint mechanisms [54], local phase decomposition mechanisms [55, 56] and seed inhibition mechanisms [57, 58] are discussed. In comparison, the analysis of the O_2_-treated sample revealed that the property changes due to the change in macroscopic coercive field is superimposed by a more severe change that cannot be explained in the sense of coercive field change. While fast switching with low activation fields, *E*_A_,　is dominant in the pristine state, the cycling induces changes that both increase the coercive field in a range similar to the N_2_-treated sample, but also significantly increases the activation field as well as shifts and broadens the characteristic switching time distribution. As recently pointed out by Buragohain et al. [34], KAI model and NLS model naturally converge at high fields due to increasingly sharp distribution of characteristic times *t*_0_, approximating the Dirac function of the KAI model. Additional file 1: Figure S1 in the Supplementary Information demonstrates the convergence of the Lorentzian distribution into a Dirac function. Small values of *w* therefore hint at switching in the sense of the KAI model, i.e., rather homogenous nuclei formation and unhindered domain wall propagation. In the present study, the values of *w* of the N_2_ annealed sample remain constant and relatively high for the cycling stages, but increase significantly for the O_2_-annealed sample. As discussed in [34], the difference between KAI and NLS switching is expected to show most significantly at low fields, i.e. for 1 < (*E*_app_/*E*_c_) < 1.5. Indeed, the difference in *w* is most significant for this range. Additionally, *t*_1_ increases for the O_2_ treated sample, pointing at increasingly inhibited nucleus formation.

We propose the following model as explanation for these observations: The atmospheric oxygen annealing step results in a reduced oxygen vacancy concentration in the film, i.e. a lower defect concentration. Importantly, the orthorhombic phase is not destabilized as evidenced by X-ray analysis in our previous study [24] and similar *P*_r_ during our electrical measurements. The o-phase stabilization is sufficiently provided by the CeO_2_ doping as well as the lattice matching with the (111)ITO bottom electrode. Epitaxial films of other phases, such as tetragonal and monoclinic, were obtained by changing the CeO_2_ concentration. So, stabilization of the desired orthorhombic phase is mainly determined by the CeO_2_ concentration. During the initial switching cycles, the lowered defect concentration in the O_2_-treated sample allows for homogeneous domain nucleus formation. This behavior can be described by KAI model, or, as demonstrated in Additional file 1: Figure S1, by low values for NLS parameter *w*. In comparison, the N_2_-treated sample is comparably defect-rich from the beginning, resulting in nucleus inhibition and inhomogeneous domain nucleus formation. This behavior is well described by the NLS model with moderate values of *w*. Continued field cycling results in generation of oxygen vacancy defects in the films. The subsequent redistribution within the film as discussed for other samples [59–61] leads to domain nucleus inhibition at the defect sites and slightly increased macroscopic coercive fields. The O_2_-treated sample is affected more significantly by the generation and redistribution of oxygen vacancies since its initial concentration is low. The samples behave nearly identical once the distributions assimilate, as seen from the similar activation fields and the NLS parameters. The fatigue property of the capacitors is not significantly influenced by this process, as the redistribution is finished after 10^3^ to 10^4^ cycles, which is far below the typically observed 10^6^ cycles until breakdown of both samples.

**Fig. 6:**
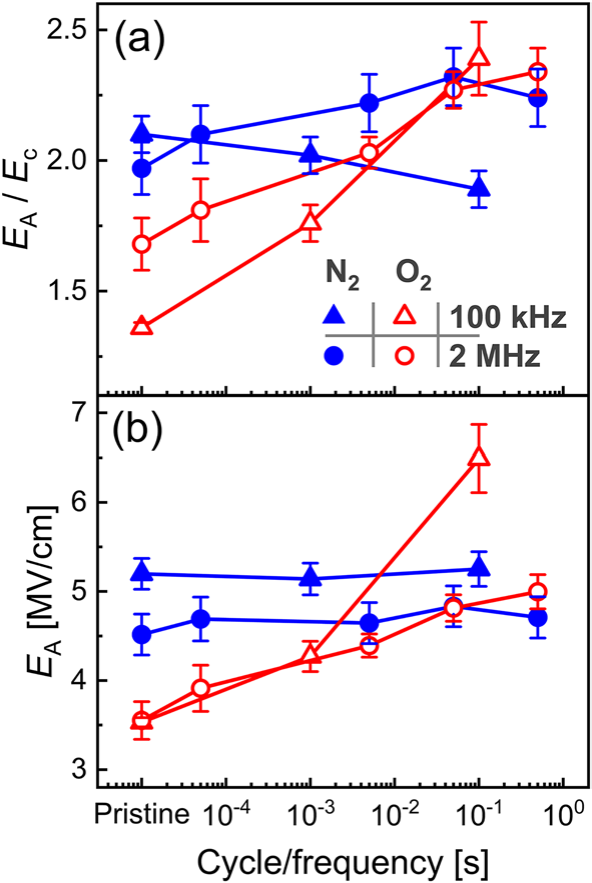
Activation field frequency dependence

As alternating fields are proposed to cause the defect generation and redistribution, significant frequency dependence is expected. Indeed, by repeating the previously described experiment on fresh capacitors and increasing the cycling frequency, *f*, from 100 kHz to 2 MHz, the shift of activation field, *E*_A_, and the ratio (*E*_A_/*E*_c_) is delayed with respect to cycle number. However, the applied field time *t*_applied_ = (*cycle*/*f* ) is expected to be the determining factor. Figure 6a and b show the ratio (*E*_A_/*E*_c_) and *E*_A_, respectively. The activation field of the N_2_-treated sample shows slight device to device variability, but *E*_A_ of both capacitors remains constant over the cycling. In contrast to that, the O_2_-treated sample shows aligning time dependence with an increasing trend as expected. The activation field values of the N_2_ samples are in good agreement with another study on epitaxial Y-doped HfO_2_ films [34], while it is around 2 to 4 times higher than reports on polycrystalline samples [36, 62]. Since *E*_c_ of polycrystalline films are typically lower, this observation holds true for the ratio (*E*_A_/*E*_c)_. The 100 kHz experiment was conducted for the opposite switching direction also, which revealed the same relationships and values and is not shown here.

The presence of oxygen vacancy defects in HfO_2_ thin films is undisputed, yet their influence on ferroelectric switching is still under debate. On the one hand, the stabilizing effect of oxygen deficiency is agreed upon [49, 62–64]. On the other hand, the influence on the switching kinetics is controversial, including reports of no direct influence [65] and switching speed enhancements [35, 64]. In the present study, we found faster switching with close to KAI model dynamics for the sample with the higher oxygen content. Yet, our results are not necessarily in contrast to other studies as several important factors are different: First, the measured effect diminishes over the period of around 10 ms field cycling (10^3^ cycles at 100 kHz), which is below typical wake-up times in polycrystalline samples [25, 26, 50, 66–68]. The phenomenon may therefore be only observable in epitaxial samples without the masking wake-up effect. Second, the highly oriented epitaxial nature of the films may be a crucial prerequisite for obtaining homogeneous switching similar to KAI dynamics. Grain boundaries and other volume defects of polycrystalline films may prohibit switching with KAI model dynamics, even when oxygen vacancy defects are excluded. Third, the stabilizing effect of oxygen vacancies in polycrystalline films [49, 62–64] implies that the vacancy concentration also changes the phase composition and remanent polarization, which may alter the switching speed simultaneously. Our precisely doped and lattice matched films showed identical structural properties, see [24], and remanent polarization, which proves that the defect concentration and the phase composition are disentangled in our samples. Lastly, the valence state of dopants in HfO_2_ may have significant impact on the ionic mobility [69]. This circumstance will require further studies, e.g. comparing epitaxial films of the popular tetravalent (Hf_0.5_Zr_0.5_)O_2_ and Si:HfO_2_ [10, 70] systems with mixed-valence dopants such as the presented Ce^3+^/Ce^4+^ and trivalent options such as Y:HfO_2_ or La:HfO_2_ [28, 34, 71].

## Conclusion

Epitaxially-grown orthorhombic Hf_0.83_Ce_0.17_O_2_ capacitors were investigated with respect to their switching dynamics at different cycling stages. While the post-deposition annealing in N_2_ and O_2_ atmosphere does not impact the constituent phase composition and the macroscopic ferroelectric properties, the sample treated in O_2_ atmosphere switches significantly faster and more uniformly than the N_2_ sample in the pristine state. As cycling continues, both samples show slight increase in coercive field. The O_2_ sample additionally transitions from a uniform switching process similar to KAI dynamics into a non-uniform region-by-region-like switching described by NLS dynamics, which is the prevalent mode in the N_2_ treated sample for all measured cycling stages. The assimilation is completed after around 10^3^ cycles at 100 kHz, which is three orders of magnitude below the typical failure. The process is explained by an oxygen vacancy defect reduction during heat treatment and gradual cycling-induced defect re-injection and redistribution.

## Supplementary Information

**Additional file 1: Fig. S1.** Distribution functions for different values of *w*in the NLS model for *t*_1_ =1 µs. In the KAI model, a single characteristic time is assumed through a Dirac function. For small values of *w*, the functions assimilate.

## Data Availability

The data of this study is available from the corresponding authors upon reasonable request.

## References

[1] K. Mistry, C. Allen, C. Auth, B. Beattie, D. Bergstrom, M. Bost, M. Brazier, M. Buehler, A. Cappellani, R. Chau, C. Choi, G. Ding, K. Fischer, T. Ghani, R. Grover, W. Han, D. Hanken, M. Hatttendorf, J. He, J. Hicks, R. Huessner, D. Ingerly, P. Jain, R. James, L. Jong, S. Joshi, C. Kenyon, K. Kuhn, K. Lee, H. Liu, J. Maiz, B. McIntyre, P. Moon, J. Neirynck, S. Pei, C. Parker, D. Parsons, C. Prasad, L. Pipes, M. Prince, P. Ranade, T. Reynolds, J. Sandford, L. Schifren, J. Sebastian, J. Seiple, D. Simon, S. Sivakumar, P. Smith, C. Thomas, T. Troeger, P. Vandervoorn, S. Williams, K. Zawadzki, “A 45nm logic technology with high-k plus metal gate transistors, strained silicon, 9 Cu interconnect layers, 193nm dry patterning, and 100% Pb-free packaging,” 2007 IEEE International Electron Devices Meeting, Vols 1 and 2, pp. 247 (2007)

[2] Spassov D, Paskaleva A, Krajewski TA, Guziewicz E, Luka G, Ivanov T (2018). Al_2_O_3_/HfO_2_ multilayer High-k Dielectric Stacks for Charge Trapping Flash Memories. Phys. Status Solidi A-Appl. Mat..

[3] T.S. Boescke, S. Govindarajan, C. Fachmann, J. Heitmann, A. Avellan, U. Schroeder, S. Kudelka, P.D. Kirsch, C. Krug, P.Y. Hung, S.C. Song, B.S. Ju, J. Price, G. Pant, B.E. Gnade, W. Krautschneider, B. Lee, R. Jammy, “Tetragonal phase stabilization by doping as an enabler of thermally stable HfO_2_ based MIM and MIS capacitors for sub 50 nm deep trench DRAM,” 2006 International Electron Devices Meeting, Vols 1 and 2, pp. 934+ (2006)

[4] Cüppers F, Menzel S, Bengel C, Hardtdegen A, von Witzleben M, Böttger U, Waser R, Hoffmann-Eifert S (2019). Exploiting the switching dynamics of HfO_2_-based ReRAM devices for reliable analog memristive behavior. APL Mater.

[5] B. Govoreanu, G.S. Kar, Y.-Y. Chen, V. Paraschiv, S. Kubicek, A. Fantini, I.P. Radu, L. Goux, S. Clima, R. Degraeve, N. Jossart, O. Richard, T. Vandeweyer, K. Seo, P. Hendrickx, G. Pourtois, H. Bender, L. Altimime, D.J. Wouters, J.A. Kittl, M. Jurczak, “10x10 nm^2^ Hf/HfO_x_ Crossbar Resistive RAM with Excellent Performance, Reliability and Low-Energy Operation,” IEDM Tech. Dig pp. 31.6.1–31.6.4 (2011)

[6] Boescke TS, Mueller J, Braeuhaus D, Schroeder U, Boettger U (2011). Ferroelectricity in Hafnium Oxide thin films. Appl. Phys. Lett.

[7] Shimizu T, Katayama K, Kiguchi T, Akama A, Konno TJ, Funakubo H (2015). Growth of epitaxial orthorhombic YO_15_-substituted HfO_2_ thin film. Appl. Phys. Lett..

[8] Richter C, Schenk T, Park MH, Tscharntke FA, Grimley ED, LeBeau JM, Zhou C, Fancher CM, Jones JL, Mikolajick T, Schroeder U (2017). Si Doped Hafnium Oxide - A ‘fragile’ ferroelectric system. Adv. Electron. Mater.

[9] Fina I, Sanchez F (2021). Epitaxial ferroelectric HfO_2_ Films: Growth, Properties, and Devices. ACS Appl. Electron. Mater.

[10] Nukala P, Antoja-Lleonart J, Wei Y, Yedra L, Dkhil B, Noheda B (2019). Direct Epitaxial Growth of Polar (1-x)HfO_2_-(x)ZrO_2_ ultrathin Films on Silicon. ACS Appl. Electron. Mater.

[11] Begon-Lours L, Mulder M, Nukala P, de Graaf S, Birkholzer YA, Kooi B, Noheda B, Koster G, Rijnders G (2020). Stabilization of phase-pure rhombohedral HfZrO_4_ in pulsed laser deposited thin films. Phys. Rev. Mater..

[12] Nukala P, Wei Y, de Haas V, Guo Q, Antoja-Lleonart J, Noheda B (2020). Guidelines for the stabilization of a polar rhombohedral phase in epitaxial Hf_0.5_Zr_0.5_O_2_ thin films. Ferroelectrics.

[13] Wei Y, Nukala P, Salverda M, Matzen S, Zhao HJ, Momand J, Everhardt AS, Agnus G, Blake GR, Lecoeur P, Kooi BJ, Iniguez J, Dkhil B, Noheda B (2018). A rhombohedral ferroelectric phase in epitaxially strained Hf_0.5_Zr_0.5_O_2_ thin films. Nat. Mater.

[14] Laudadio E, Stipa P, Pierantoni L, Mencarelli D (2022). Phase Properties of different HfO_2_ polymorphs: a DFT-Based study. Crystals.

[15] Zhang Y, Yang Q, Tao L, Tsymbal EY, Alexandrov V (2020). Effects of strain and Film Thickness on the Stability of the Rhombohedral phase of HfO_2_. Phys. Rev. Appl.

[16] Mimura T, Shimizu T, Uchida H, Funakubo H (2020). Room-temperature deposition of ferroelectric HfO_2_-based films by the sputtering method. Appl. Phys. Lett.

[17] Yoong HY, Wu H, Zhao J, Wang H, Guo R, Xiao J, Zhang B, Yang P, Pennycook SJ, Deng N, Yan X, Chen J (2018). Epitaxial ferroelectric Hf_0.5_Zr_0.5_O_2_ thin Films and their implementations in memristors for brain-inspired Computing. Adv. Funct. Mater.

[18] Shimura R, Mimura T, Shimizu T, Tanaka Y, Inoue Y, Funakubo H (2020). Preparation of near-1-mu m-thick {100}-oriented epitaxial Y-doped HfO_2_ ferroelectric films on (100)Si substrates by a radio-frequency magnetron sputtering method. J. Ceram. Soc. Jpn.

[19] Kiguchi T, Nakamura S, Akama A, Shiraishi T, Konno TJ (2016). Solid state epitaxy of (Hf,Zr)O_2_ thin films with orthorhombic phase. J. Ceram. Soc. Jpn.

[20] Mimura T, Shimizu T, Kiguchi T, Akama A, Konno TJ, Katsuya Y, Sakata O, Funakubo H (2019). Effects of heat treatment and in situ high-temperature X-ray diffraction study on the formation of ferroelectric epitaxial Y-doped HfO_2_ film. Jpn J. Appl. Phys.

[21] Chernikova AG, Kozodaev MG, Negrov DV, Korostylev EV, Park MH, Schroeder U, Hwang CS, Markeev AM (2018). Improved ferroelectric switching endurance of La-Doped Hf_0.5_Zr_0.5_O_2_ thin Films. ACS Appl. Mater. Interfaces.

[22] Shiraishi T, Choi S, Kiguchi T, Shimizu T, Uchida H, Funakubo H, Konno TJ (2018). Fabrication of ferroelectric Fe doped HfO_2_ epitaxial thin films by ion-beam sputtering method and their characterization. Jpn. J. Appl. Phys..

[23] Shiraishi T, Choi S, Kiguchi T, Shimizu T, Funakubo H, Konno TJ (2019). Formation of the orthorhombic phase in CeO_2_-HfO_2_ solid solution epitaxial thin films and their ferroelectric properties. Appl. Phys. Lett.

[24] Hirai K, Shiraishi T, Yamaoka W, Tsurumaru R, Inoue Y, Funakubo H (2022). Composition dependence of ferroelectric properties in (111)-oriented epitaxial HfO_2_-CeO_2_ solid solution films. Jpn J. Appl. Phys.

[25] Fields SS, Smith SW, Ryan PJ, Jaszewski ST, Brummel IA, Salanova A, Esteves G, Wolfley SL, Henry MD, Davids PS, Ihlefeld JF (2020). Phase-exchange-driven Wake-Up and fatigue in Ferroelectric Hafnium Zirconium Oxide Films. ACS Mat. Interf.

[26] Kim HJ, Park MH, Kim YJ, Lee YH, Moon T, Do Kim K, Hyun SD, Hwang CS (2016). A study on the wake-up effect of ferroelectric Hf_0.5_Zr_0.5_O_2_ films by pulse-switching measurement. Nanoscale.

[27] S. Shibayama, L. Xu, S. Migita, A. Toriumi, “Study of wake-up and fatigue properties in doped and undoped ferroelectric HfO_2_ in conjunction with piezo-response force microscopy analysis,” 2016 IEEE Symposium On Vlsi Technology (2016)

[28] Li X, Li C, Xu Z, Li Y, Yang Y, Hu H, Jiang Z, Wang J, Ren J, Zheng C, Lu C, Wen Z (2021). Ferroelectric Properties and polarization fatigue of La:HfO_2_ Thin-Film Capacitors. Phys. Status Solidi-Rapid Res. Lett.

[29] Song T, Bachelet R, Saint-Girons G, Solanas R, Fina I, Sanchez F (2020). Epitaxial ferroelectric La-Doped Hf_0.5_Zr_0.5_O_2_ thin Films. ACS Appl. Electron. Mater.

[30] Lyu J, Song T, Fina I, Sanchez F (2020). High polarization, endurance and retention in sub-5 nm Hf_0.5_Zr_0.5_O_2_ films. Nanoscale.

[31] Rabe KA, Dawber M, Lichtensteiger C, Ahn CH, Triscone JM (2007). Modern physics of ferroelectrics: essential background. Top. Appl. Phys.

[32] Merz WJ (1954). Domain formation and domain wall motions in ferroelectric BaTiO_3_ single crystals. Phys. Rev. USA.

[33] X. Lyu, M. Si, P.R. Shrestha, K.P. Cheung, P.D. Ye, “First Direct Measurement of Sub-Nanosecond Polarization Switching in Ferroelectric Hafnium Zirconium Oxide,” *IEEE*, 2019, pp. 15.2.1–15.2.4 10.1063/1.5098786 PMC11194797 38915734

[34] Buragohain P, Erickson A, Mimura T, Shimizu T, Funakubo H, Gruverman A (2022). Effect of Film microstructure on domain nucleation and intrinsic switching in ferroelectric Y:HfO_2_ Thin Film Capacitors. Adv. Funct. Mater.

[35] Lee K, Park K, Lee HJ, Song MS, Lee KC, Namkung J, Lee JH, Park J, Chae SC (2021). Enhanced ferroelectric switching speed of Si-doped HfO_2_ thin film tailored by oxygen deficiency. Sci. Rep.

[36] Materano M, Lomenzo PD, Mulaosmanovic H, Hoffmann M, Toriumi A, Mikolajick T, Schroeder U (2020). Polarization switching in thin doped HfO_2_ ferroelectric layers. Appl. Phys. Lett..

[37] Ishibashi Y, Takagi Y (1971). Ferroelectric domain switching. J. Phys. Soc. Jpn..

[38] Avrami M (1939). Kinetics of Phase Change I: General Theory. J. Chem. Phys.

[39] Kolmogorov AN (1937). On the statistical theory of metal crystallisation. Izv. Akad. Nauk SSSR Ser. Mat..

[40] Zhukov S, Genenko YA, Hirsch O, Glaum J, Granzow T, von Seggern H (2010). Dynamics of polarization reversal in virgin and fatigued ferroelectric ceramics by inhomogeneous field mechanism. Phys. Rev. B: Condens. Matter.

[41] Buyantogtokh B, Gaddam V, Jeon S (2021). Effect of high pressure anneal on switching dynamics of ferroelectric hafnium zirconium oxide capacitors. J. Appl. Phys.

[42] Hoffmann M, Pešic M, Chatterjee K, Khan A.I, Salahuddin S, Slesazeck S, Schroeder U, Mikolajick T (2016). Direct Observation of negative capacitance in Polycrystalline Ferroelectric HfO_2_. Adv. Funct. Mater..

[43] Buragohain P, Richter C, Schenk T, Lu H, Mikolajick T, Schroeder U, Gruverman A (2018). Nanoscopic studies of domain structure dynamics in ferroelectric La: HfO2 capacitors. Appl. Phys. Lett..

[44] X. Du, I. Chen, “Frequency spectra of fatigue of PZT and other ferroelectric thin films,” Materials Research Society Symposium Proceedings, pp. 311–316, (1998)

[45] Mulaosmanovic H, Ocker J, Mueller S, Schroeder U, Mueller J, Polakowski P, Flachowsky S, van Bentum R, Mikolajick T, Slesazeck S (2017). Switching kinetics in Nanoscale Hafnium Oxide Based Ferroelectric Field-Effect Transistors. ACS Appl. Mater. Interfaces.

[46] Kim Y, Han H, Lee W, Baik S, Hesse D, Alexe M (2010). Non-Kolmogorov-Avrami-Ishibashi Switching Dynamics in Nanoscale Ferroelectric Capacitors. Nano Lett.

[47] Hyun SD, Park HW, Kim YJ, Park MH, Lee YH, Kim HJ, Kwon YJ, Moon T, Kim KD, Lee YB, Kim BS, Hwang CS (2018). Dispersion in ferroelectric switching performance of Polycrystalline Hf_0.5_Zr_0.5_O_2_ thin Films. ACS Appl. Mater. Interfaces.

[48] Tagantsev AK, Stolichnov I, Setter N, Cross JS, Tsukada M (2002). Non-Kolmogorov-Avrami switching kinetics in ferroelectric thin films. Phys. Rev. B (Condensed Matter Mater. Physics) USA.

[49] Ryu TH, Min DH, Yoon SM (2020). Comparative studies on ferroelectric switching kinetics of sputtered Hf_0.5_Zr_0.5_O_2_ thin films with variations in film thickness and crystallinity. J. Appl. Phys.

[50] Lee TY, Lee K, Lim HH, Song MS, Yang SM, Yoo HK, Suh DI, Zhu Z, Yoon A, MacDonald MR, Lei X, Jeong HY, Lee D, Park K, Park J, Chae SC (2019). Ferroelectric polarization-switching Dynamics and Wake-Up Effect in Si-Doped HfO_2_. ACS Appl. Mater. Interfaces.

[51] Materano M, Lomenzo PD, Kersch A, Park MH, Mikolajick T, Schroeder U (2021). Interplay between oxygen defects and dopants: effect on structure and performance of HfO_2_-based ferroelectrics. Inorg. Chem. Front.

[52] Mueller J, Schroeder U, Boescke TS, Mueller I, Boettger U, Wilde L, Sundqvist J, Lemberger M, Kuecher P, Mikolajick T, Frey L (2011). Ferroelectricity in yttrium-doped hafnium oxide. J. Appl. Phys.

[53] Warren WL, Dimos D, Tuttle BA, Pike GE, Schwartz RW, Clews PJ, McIntyre DC (1995). Polarization suppression in Pb(Zr,Ti)O_3_ thin films. J. Appl. Phys.

[54] Grossmann M, Lohse O, Bolten D, Boettger U, Waser R, Hartner W, Kastner M, Schindler G (2000). Lifetime estimation due to imprint failure in ferroelectric SrBi_2_Ta_2_O_9_ thin films. Appl. Phys. Lett.

[55] Lou XJ, Zhang M, Redfern SAT, Scott JF (2006). Local phase decomposition as a cause of polarization fatigue in ferroelectric thin films. Phys. Rev. Lett..

[56] Lou XJ, Zhang M, Redfern SAT, Scott JF (2007). Fatigue as a local phase decomposition: a switching-induced charge-injection model. Phys. Rev. B..

[57] Colla EL, Tagantsev AK, Taylor DV, Kholkin AL (1997). Fatigued state of the Pt-PZT-Pt system. Int Ferroelectr.

[58] Colla EL, Seungbum-Hong DV, Taylor AK, Tagantsev N, Setter, Kwangsoo-No (1998). Direct observation of region by region suppression of the switchable polarization (fatigue) in pb(zr,Ti)O_3_ thin film capacitors with pt electrodes. Appl. Phys. Lett.

[59] Starschich S, Menzel S, Böttger U (2016). Evidence for oxygen vacancies movement during wake-up in ferroelectric hafnium oxide. Appl. Phys. Lett..

[60] Meyer R, Liedtke R, Waser R (2005). Oxygen vacancy migration and time-dependent leakage current behavior of Ba_0.3_Sr_0.7_TiO_3_ thin films. Appl. Phys. Lett.

[61] Scott JF, Dawber M (2000). Oxygen-vacancy ordering as a fatigue mechanism in perovskite ferroelectrics. Appl. Phys. Lett.

[62] Choi SN, Moon SE, Yoon SM (2019). Film thickness-dependent ferroelectric polarization switching dynamics of undoped HfO_2_ thin films prepared by atomic layer deposition. Ceram. Int.

[63] Pal A, Narasimhan VK, Weeks S, Littau K, Pramanik D, Chiang T (2017). Enhancing ferroelectricity in dopant-free hafnium oxide. Appl. Phys. Lett.

[64] Yoon SJ, Na SY, Moon SE, Yoon SM (2019). Polarization switching kinetics of the ferroelectric Al-doped HfO_2_ thin films prepared by atomic layer deposition with different ozone doses. J. Vac. Sci. Technol. B..

[65] Shimizu T, Yokouchi T, Oikawa T, Shiraishi T, Kiguchi T, Akama A, Konno TJ, Gruverman A, Funakubo H (2015). Contribution of oxygen vacancies to the ferroelectric behavior of Hf_0.5_Zr_0.5_O_2_ thin films. Appl Phys Lett..

[66] Chun MC, Park S, Park S, Park Gy, Kim MJ, Cho Y, Kang BS (2020). Effect of wake-up on the polarization switching dynamics of Si doped HfO_2_ thin films with imprint. J. Alloy Compd.

[67] Park MH, Kim HJ, Kim YJ, Lee YH, Moon T, Kim KD, Hyun SD, Fengler F, Schroeder U, Hwang CS (2016). Effect of Zr Content on the Wake-Up Effect in Hf_1–x_Zr_x_O_2_ Films. ACS Appl. Mater. Interfaces.

[68] Zhou D, Xu J, Li Q, Guan Y, Cao F, Dong X, Mueller J, Schenk T, Schroeder U (2013). Wake-up effects in Si-doped hafnium oxide ferroelectric thin films. Appl. Phys. Lett.

[69] Krishnamurthy R, Yoon Y, Srolovitz D, Car R (2004). Oxygen diffusion in yttria-stabilized zirconia: a new simulation model. J. Am. Ceram. Soc.

[70] Ali T, Polakowski P, Riedel S, Buettner T, Kaempfe T, Rudolph M, Paetzold B, Seidel K, Loehr D, Hoffmann R, Czernohorsky M, Kuehnel K, Thrun X, Hanisch N, Steinke P, Calvo J, Mueller J (2018). Silicon doped hafnium oxide (HSO) and hafnium zirconium oxide (HZO) based FeFET: A material relation to device physics. Appl. Phys. Lett..

[71] Schroeder U, Richter C, Park MH, Schenk T, Pesic M, Hoffmann M, Fengler FPG, Pohl D, Rellinghaus B, Zhou C, Chung C, Jones JL, Mikolajick T (2018). Lanthanum-Doped Hafnium Oxide: a robust ferroelectric material. Inorg. Chem.

