# Supplementary material for: On the switching dynamics of epitaxial ferroelectric CeO2–HfO2 thin film capacitors
Source: Nano Converg. 2022 Dec 14;9:56. doi: 10.1186/s40580-022-00344-4 (PMC9751238; doi:10.1186/s40580-022-00344-4)
Supplement: Supplementary file 1 — Additional file 1: Fig. S1. Distribution functions for different values of win the NLS model for t1 =1 µs. In the KAI model, a single characteristic time is assumed through a Dirac function. For small values of w, the functions assimilate. [file 40580_2022_344_MOESM1_ESM.docx]

Additional file 1: On the switching dynamics of epitaxial ferroelectric CeO_2_ – HfO_2_ thin film capacitors

**Felix Cüppers^1^*, Koji Hirai^1^, and Hiroshi Funakubo^1^***

^1^Department of Materials Science and Engineering, Tokyo Institute of Technology, Yokohama 226-8502, Japan

E-mail: funakubo.h.aa@m.titech.ac.jp , cueppers.f.aa@gmail.com


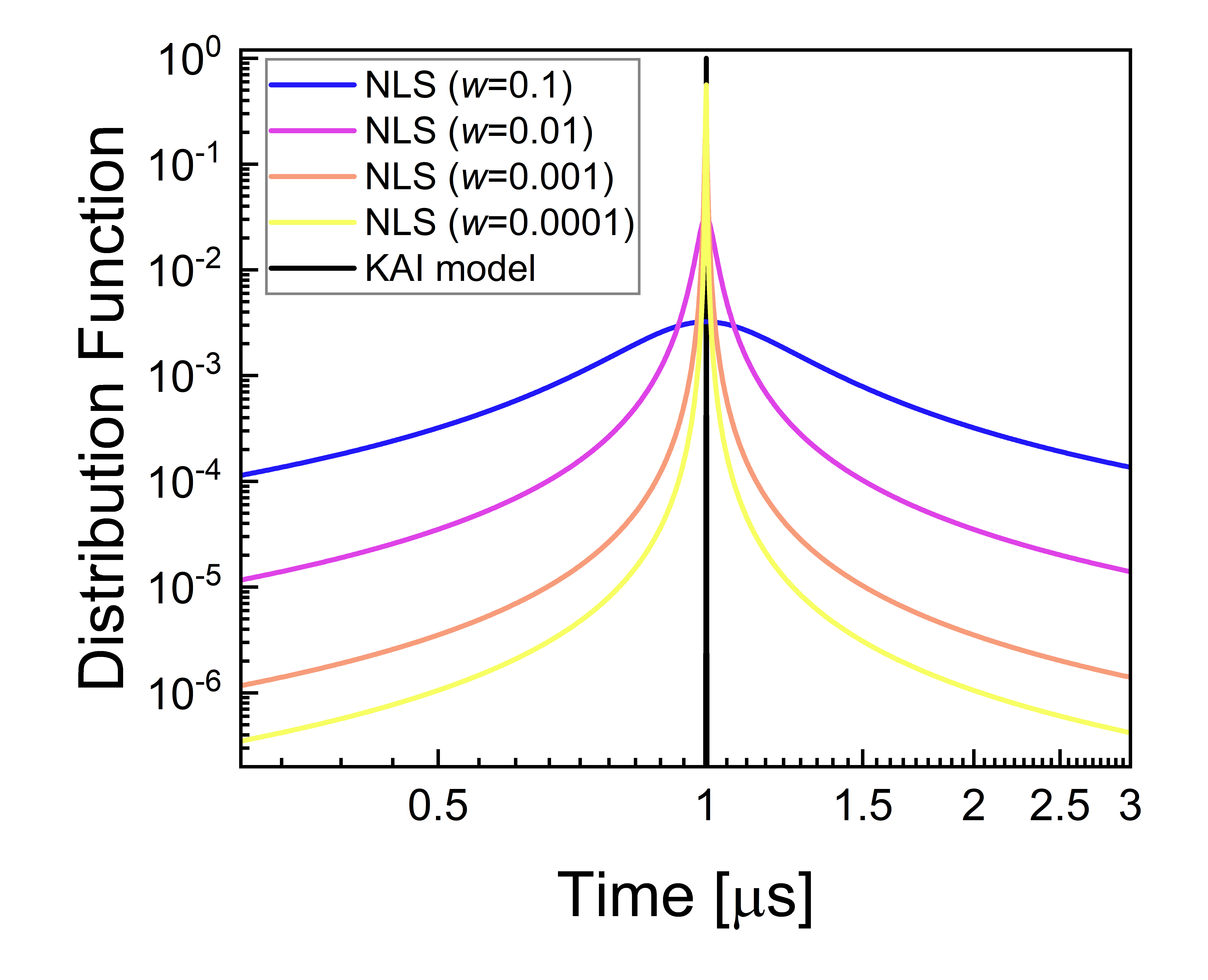


**Fig. S1.** Distribution functions for different values of *w* in the NLS model for *t*_1_ = 1 µs. In the KAI model, a single characteristic time is assumed through a Dirac function. For small values of *w*, the functions assimilate.
